# Gradients of Variation in the At-Vessel Mortality Rate between Twelve Species of Sharks and Skates Sampled through a Fishery-Independent Trawl Survey in the Asinara Gulf (NW Mediterranean Sea)

**DOI:** 10.3390/biology12030363

**Published:** 2023-02-24

**Authors:** Umberto Scacco, Tomaso Fortibuoni, Matteo Baini, Gianluca Franceschini, Dario Giani, Margherita Concato, Cristina Panti, Alessia Izzi, Michela Angiolillo

**Affiliations:** 1 National Centre of Laboratories-Biology, Italian Institute for Environmental Protection and Research (ISPRA), Via di Castel Romano 100, 00128 Rome, Italy; 2 Department of Bio Ecological Sciences, University of Tuscia, Largo dell’Università snc, 01100 Viterbo, Italy; 3 Area for the Conservation, Management and Sustainable Use of Fish Stocks and National Marine Aquatic Resources, Italian Institute for Environmental Protection and Research (ISPRA), 30015 Chioggia, Italy; 4 Department of Physical Sciences, Earth and Environment, University of Siena, Via P.A. Mattioli, 4, 53100 Siena, Italy; 5 Area for the Protection of Biodiversity, Habitats and Protected Marine Species, Italian Institute for Environmental Protection and Research (ISPRA), Via Vitaliano Brancati 60, 00144 Rome, Italy

**Keywords:** elasmobranchs, at-vessel mortality, vitality rate, bycatch, trawl fishing, Mediterranean Sea

## Abstract

**Simple Summary:**

The impact of human activities on marine environments is driving many elasmobranch species toward the brink of extinction. Fishing activities play the most important role in causing the mortality of these animals and the substantial decrease observed in their populations. In this context, we aimed to measure species resistance to catch of poorly selective fishing gear, such as trawl, and investigate the patterns of variation found in the sample. Fishery-independent standardized data indicated that small-sized deepwater sharks are the most affected by stress due to trawl catch. On the contrary, large and coastal species, particularly skates, appeared more resistant to trawl capture. Overall, the at-vessel mortality rate of the studied species results from the intermingled effect of inhabited depth, species type, and fish size. The information provided can help refine best practices to reduce direct and indirect fishing mortality of the studied species in trawling activities.

**Abstract:**

Elasmobranchs are priority species for conservation due to their rapid decline determined by the unbalanced struggle between a fragile bio-ecology and strong anthropogenic impacts, such as bycatch from professional fishing. In this context, measuring species resistance to catch of poorly selective gear is of paramount importance. During June–October 2022, five experimental fishing campaigns were carried out in the Asinara Gulf (northern Sardinia) through 35 geographically and bathymetrically representative hauls of an area between 30 and 600 m in depth. Skates prevailed over sharks in the number of species, with seven and five species, respectively. We first evaluated the status of each individual with respect to stress due to the trawl’s catch using a three-graded scale. We also recorded individual biometrics (total and disk length, weight and sex, and maturity for males) on board by implementing the best practices in manipulating individuals for physiological recovery and release at sea. After capture, skates resulted in generally better conditions than sharks, although deepwater species of both groups exhibited a worse state than coastal species. The estimated vitality rates also depended on the size of the individuals. This work provides standardized data on the intermingled effect of size, species type, and inhabited depth on the resistance response of some elasmobranch species against capture by trawl fishery activities.

## 1. Introduction

Elasmobranchs are priority species for conservation due to their rapid decline determined by the unbalanced struggle between fragile bio-ecology and heavy and increasing anthropogenic impacts [1,2,3,4,5,6,7]. Among these anthropogenic stressors, targeted and untargeted fisheries represent the most relevant threat to cartilaginous fish populations [8,9], as well as habitat degradation and climate change [10,11,12]. The decline of cartilaginous fish populations is altering the marine food webs dramatically, as these animals are high-trophic level, even top predator, species with a fundamental role in maintaining balanced biodiversity in marine ecosystems [2].

Fishing mortality varies considerably among elasmobranch species as fishing gear and métiers vary. For instance, pelagic longlines represent the main threat to pelagic sharks and rays in the world’s oceans [13,14,15,16,17], including the Mediterranean areas [3,18,19]. Fixed gill and trammel nets tend to catch a large number of sharks and rays [20,21,22,23,24,25], as well as drift nets [26,27]. Small-scale fishery impacts cartilaginous coastal-dependent species, as it usually uses passive fishing gears within coastal waters [28,29,30,31].

Poorly selective and active and semi-active gear [32,33], such as trawl (demersal and pelagic) and purse seine, are the fisheries that most threaten the populations of elasmobranch species. These fisheries exhibit high fishing mortality for the mixture of demersal and bathy pelagic bycatch species in the world’s oceans and the Mediterranean Sea [34,35,36,37,38,39,40,41]. Finally, the demersal trawl is the most dangerous fishing gear for cartilaginous fish compared to other fishing gear [42]. The multi-specific nature of elasmobranch bycatch in trawl fishery is due to the use of poorly selective gear that can vary in direct interaction with the seabed [33,43,44].

The biological responsiveness of a species to fishing pressure depends on two species-specific properties, resilience and resistance [45], given the fishing gear used.

On the one hand, resilience acts at the population level, and it represents the ability of a species to re-establish a condition of demographic balance when steady fishing mortality has significantly altered it within the population [45]. Elasmobranchs are low-resilience species, given their slow growth, delayed maturity, and low fecundity rates, making them particularly vulnerable and slow to recover from overfishing [46,47].

On the other hand, resistance is the average individual response of a species to stress caused by a disturbance, such as capture by a given fishing gear [45,48]. It can be measured by at-vessel mortality (AVM) [49], i.e., the average probability that an individual is alive or dead following an intense pulse disturbance such as capture and haul up to the vessel deck. In contrast to low resilience, cartilaginous fish show high resistance [50] instead, as higher resistance is a common trait of k-selected compared to r-selected species [51]. Synoptically, pulse and more intense disturbances trigger more rapid impacts on species resistance than steady disturbances that tend to trigger resilience dynamics [48].

AVM depends on a variety of factors in elasmobranchs [52]. It is influenced by both biological attributes (species, size, sex, and mode of gill ventilation) and other factors associated with capture (gear type, soak time, catch mass and composition, handling practices, and the degree of variation in the abiotic parameters) [52]. Physiological stress induced by the capture is demonstrated by the alteration of typical blood markers, as shown by several field studies [53,54]. AVM can be particularly high in poorly selective and active fishing gear due to the severe conditions the animals experience during capture in these fisheries [42,52].

In this context, it is of priority to measure and compare the resistance to capture by poorly selective fishing gear of different elasmobranch species, particularly in the Mediterranean Sea, where these studies are scarce [52]. Knowing this information helps refine best practices to reduce direct and indirect fishing mortality of elasmobranch species through the release in good condition of individuals caught back to the sea. The release of healthy individuals after capture can improve the resilience capacity of populations of different bycatch species by reducing the impact fishing has on them [35,45].

This is particularly significant in areas that still exhibit a high level of biodiversity and have the potential to be a refuge for elasmobranch species. Located in northern Sardinia (Italy), the Asinara Gulf is a marine area of relevant environmental value [55,56,57,58], representing a hot spot for marine species’ biodiversity [59]. It shows a high heterogeneity of the seabed, with strong variations in bathymetry (the presence of shoals and canyons) [60] and important habitats, such as *Posidonia oceanica* meadows [61], coralligenous [62,63], sandy, muddy, and rocky grounds [64] favoring general species richness [65,66]. The presence of three protected areas surrounding the study area is emblematic of these peculiarities: the Asinara National Park and the Marine Protected Area of S. Teresa on the west and east ends of the Gulf, respectively, and the Scandola marine reserve [67] far north in neighboring Corsica.

Excluding the numerous fishery-interdicted areas [66], the Gulf represents a fishing ground for trawl and, in particular, for small-scale fisheries. The distribution of fishing efforts between the two fishing segments is in line with the data available for Sardinian waters [68]. Demersal trawl represents a small fraction (9%) compared to passive polyvalent gear (91%) based on the number of fishing vessels. However, percentages reverse if the gross tonnage is considered (61% trawls and 38% polyvalent passive gear) [68]. The Mediterranean cod *Merluccius merluccius*, the red mullets *Mullus* sp., and the musky octopuses *Eledone* sp. are the target species in the coastal demersal trawl fishery.

In contrast, the Norway lobster *Nephrops norvegicus*, the rose shrimp *Parapenaeus longirostris,* and the red shrimps *Aristeus antennatus* and *Aristaeomorfa foliacea* represent the species targeted in deepwater demersal trawlers of the area [68]. The local small-scale fishery includes a large array of fishing gear targeting high commercial value species, such as the spiny and European lobsters *Palinurus elephas* and *Homarus gammarus* (trammel nets and pots, respectively), fish species belonging to Sparidae and Sciaenidae families (trammel and gill nets, respectively), the common octopus *Octopus vulgaris* (pots), and the swordfish *Xiphias gladius* and the red scorpionfish *Scorpaena* sp. (pelagic and bottom longlines, respectively) [66]. Catalano et al. [69,70] provided a focused description of the elasmobranch species present in the surrounding area of Asinara National Park, and a list of elasmobranchs is updated yearly by the Mediterranean International Trawl Survey (MEDITS) program for the Sardinian waters (geographical sampling area (GSA) 11) [71].

In the present work, we measured the species-specific resistance to demersal trawl capture in twelve shark and skate species typical of the bycatch of coastal and deep Mediterranean trawling activities through a fishery-independent scientific survey. This work investigates the effect of fish size, species type, and capture depth on the gradients of variation observed in the AVM rate among the species studied.

## 2. Materials and Methods

### 2.1. Sampling Design and On-Board Activities

**Figure 1:**
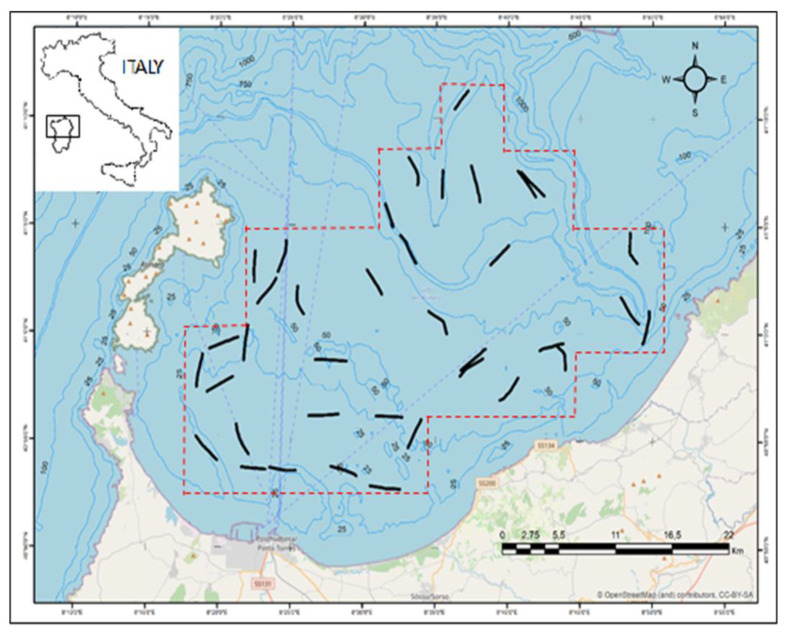
The sampling area (Asinara Gulf, NW Sardinia, Italy) was explored during a fishery-independent trawl survey during June–October 2022. Dark lines represent the 35 hauls, plus 2 repeated (due to technical problems: overlapping dark lines) hauls, performed and distributed within the grid of 5 km × 5 km squares (red dotted lines) selected for fishing operations.

A fishery-independent trawl survey was carried out in the Asinara Gulf (Sardinia, north-western Mediterranean Sea) in a preselected area of 650 km^2^ as bathymetrically and geographically representative as possible of the Asinara Gulf (Figure 1). Thirty-five sampling stations were equally distributed across a 5 × 5 km square grid covering the area selected for sampling operations. Therefore, 35 hauls (plus two haul repetitions for invalid sampling due to technical problems that occurred) were carried out as close as possible to the selected sampling stations through five fishing campaigns between June and October 2022 (Figure 1). The sampling stations and haul routes lightly differed from the fishing stations selected, as the hauls were carried out where trawl fishing was practicable, based on the information provided to us by the captain (presence of objects harmful to the net or bottom morphology not correct for trawling).

Fishing operations involved the Saturno trawl fishing boat (gross tonnage 50 tons, engine power 500 KW, and overall boat length 21 m) using a diamond (coded mesh size 40 mm) commercial trawl net (23 × 2.5 × 40 m). Thirty minutes was the standardized haul duration, and the hauls were carried out between 7:00 am and 5:00 pm. Fishing data were recorded for each haul, including start and end geographical coordinates, wind speed, sea state, and global positioning system (GPS) tracks. All of the elasmobranch specimens caught were photo-recorded and identified at the lowest possible taxonomic level considering the morphological diagnostic characters reported in the available literature and taxonomical guides [72,73].

The vitality rate was assessed through a three-graded scale and classified as (i) dead, (ii) inactive, and (iii) active (Table 1), adapted from the scale of [74]. Biometrics, such as sex, total, and disk length, with 1 cm precision, and approximate weight, were also collected. Length and weight measurements were taken by a 1 cm graduated tape meter and a dynamometer (0.1–50 kg), respectively. These operations were conducted as promptly and gently as possible to speed up the immersion of captured elasmobranch specimens into tanks (1 m × 1 m × 0.5 m) flushed with seawater [75,76], following the best manipulating practices [42,77]. This was carried out to provide specimens with oxygenated water to favor their recovery from stress due to the catch as best as possible before releasing them at sea. Once the overall assessment had finished, we released each specimen at sea after about 30 min of immersion in the recovery tanks.

### 2.2. Data Analysis

The species were coded using the initial letter and the two first letters of the genus and the species name, respectively (Table 2). The full name of the species with authority and corresponding codes is reported at the beginning of the Results section.

Because the data were counts, we used contingency tables and associated chi-square tests to investigate the size effect on the vitality rate within each species. The data were expressed as the number of individuals grouped into size intervals by the vitality rate. For this purpose, we divided the species length ranges into three (small, medium, and large) or two (medium and large) equal intervals based on the length distribution of each species (Table 2). Therefore, we organized the contingency tables with the columns as vitality rates (active, inactive, and dead or active and inactive) and the rows as size groups to obtain 3 × 3, 3 × 2, and 2 × 2 tables according to the species.

We used Friedman’s nonparametric ANOVA to investigate the differences in the importance of vitality rates (expressed as relative percentages of individuals grouped into vitality rates within each species) across species (with species as rows and vitality rates as columns). The associated Kendall’s concordance coefficient was used to check for similarity/dissimilarity in ranking the importance of vitality rates across species. As a post-hoc analysis, we used a 9 × 3 contingency table and an associated chi-square test to identify groups (expressed as the number of individuals by species and vitality rate) that contributed the most variance to the total variation between the observed and expected values of the abundance in each group.

Finally, we used principal component analysis (PCA) to study the effect of the mean depth of capture, fish size, and species type, used as supplementary variables, on the vitality rates of individuals of each species, used as active variables. To perform the analysis, we transformed the categorical variables (both active and supplementary) into discrete and ordinal variables by associating numerical values with the categories. Specifically, we associated 1, 2, and 3 with active, inactive, and dead specimens, respectively, and 1 or 2 in the case where the individuals were sharks or rays, respectively. Size and capture mean depth were used as continuous descriptors. The capture mean depth was calculated as a weighted average (multiplied) over the number of individuals per haul.

Species with unique and/or sparse observations (Table 2) were excluded from all analyses, except for PCA, as a descriptive value multivariate statistical technique. The relative and absolute catch per unit effort (CPUE) of each species was calculated as the number of individuals caught by fishing hour upon the total number of hauls performed and upon positive hauls alone, respectively. The mean absolute CPUE by haul was also provided for each species. All statistical analyses were performed with STATISTICA software version 7.0 [78].

## 3. Results

### 3.1. General Data

**Figure 2:**
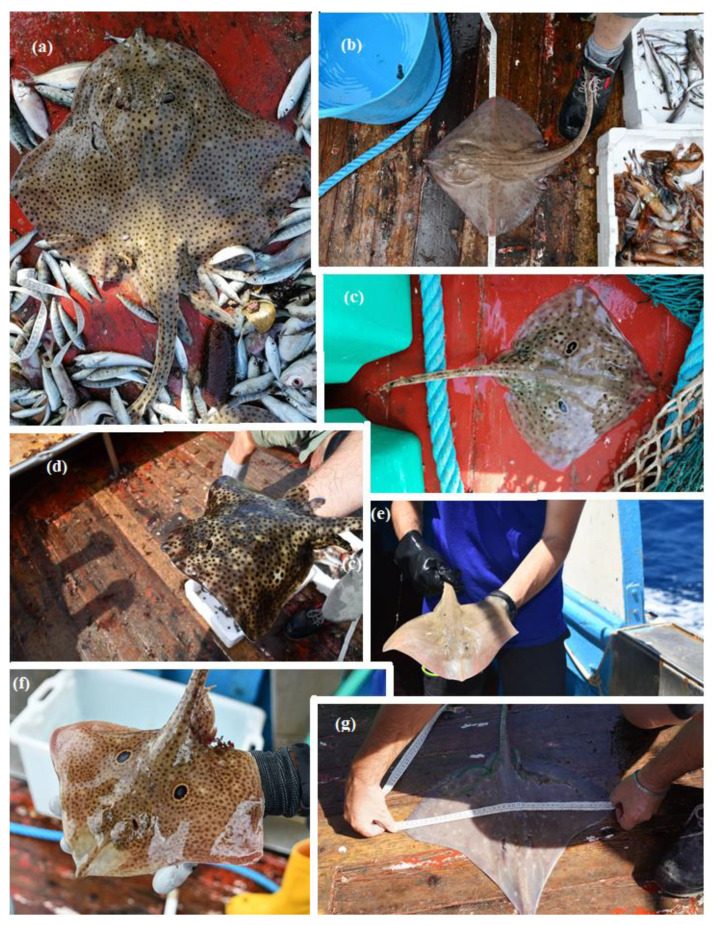
Images of the skate species sampled during a fishery-independent trawl survey in the Asinara Gulf during June–October 2022. (**a**) *Raja brachyura*, (**b**) *Leucoraja circularis*, (**c**) *Raja polystigma*, (**d**) *Raja montagui*, (**e**) *Leucoraja fullonica*, (**f**) *Raja miraletus,* and (**g**) *Dipturus oxyrinchus*.

The bathymetric interval explored during the scientific trawl survey spanned between the shallow coastal shelf and the bathyal grounds (Table 3). Overall, we sampled twelve species of elasmobranchs (Table 3), in particular:

*1.*  Five species of sharks, such as the picked dogfish Squalus acanthias Linnaeus, 1758 (SAC), the smooth-hound shark Mustelus mustelus (Linnaeus, 1758) (MMU), the velvet belly Etmopterus spinax (Linnaeus, 1758) (ESP), the small-spotted catshark Scyliorhinus canicula (Linnaeus, 1758) (SCA), and the black-mouth catshark Galeus melastomus Rafinesque, 1810 (GME) (Figure S1);
*2.*  Seven species of skates, such as the shagreen ray Leucoraja fullonica (Linnaeus, 1758) (LFU), the sandy ray L. circularis (Couch, 1838) (LCI), the spotted ray Raja montagui Fowler, 1910 (RMO), the blonde ray R. brachyura Lafont, 1873 (RBR), the speckled ray R. polystigma Regan, 1923 (RPO), the brown ray R. miraletus Linnaeus, 1758 (RMI), and the long-nosed skate Dipturus oxyrinchus (Linnaeus, 1758) (DOX) (Figure 2).

**Table 3:** Sample size (n.)**,** size range, and mean size as total length (TL in sharks) and disk width (DW in skates), haul depth range and haul mean depth (weighed on the number of individuals by haul), and mean catch per unit effort (CPUE) (as the number of individuals caught per fishing hour) by twelve species of elasmobranchs arranged in alphabetical order and sampled through a fishery-independent trawl survey in the Asinara Gulf during June–October 2022. Means are provided with their standard deviation (SD.) Species codes *DOX*, *ESP*, *GME*, *LCI*, *LFU*, *MMU*, *RBR*, *RMI*, *RMO*, *RPO*, *SAC*, and *SCA* refer to *Dipturus oxyrinchus*, *Etmopterus spinax*, *Galeus melastomus*, *Leucoraja circularis*, *L. fullonica*, *Mustelus mustelus*, *Raja brachyura*, *R. miraletus*, *R. montagui*, *R. polystigma*, *Scyliorhinus canicula,* and *Squalus acanthias*, respectively.

Species	n.	Size Range (cm)	Mean Size (TL or DW) ± SD	Haul Depth Range (m)	Haul Mean Depth (m) ± SD	Mean Absolute CPUE (ind/h) ± SD	Relative CPUE (ind/h)
*DOX*	20	24–62	43.25 ± 11.73	270–360	324.2 ± 18.7	13.3 ± 7.7	1.14
*ESP*	42	12–42	24.92 ± 7.08	550–600	583.9 ± 12.1	42.0 ± 12.0	2.40
*GME*	721	10–49	21.81 ± 6.86	133–600	407.6 ± 62.1	206 ± 281.1	41.2
*LCI*	2	42–47	44.5 ± 2.50	200–210	205.0	4.0	0.11
*LFU*	2	30–45	37.5 ± 7.50	70–200	135.0	4.0	0.11
*MMU*	1	41	na	33–35	34.0	2.0	0.06
*RBR*	16	24–57	38.31 ± 9.31	33–360	209.4 ± 107.7	5.3 ± 2.9	0.91
*RMI*	35	13–41	26.57 ± 7.56	33–136	68.0 ± 12.0	7.0 ± 4.2	2.00
*RMO*	12	9–47	23.50 ± 14.77	42–210	159.7 ± 60.6	6.0 ± 4.7	0.68
*RPO*	36	15–55	21.32 ± 10.36	43–350	140.8 ± 61.0	7.2 ± 9.26	2.06
*SCA*	714	10–50	23.53 ± 9.75	33–480	147.9 ± 89.7	54.8 ± 59.4	40.8
*SAC*	7	45–72	58.14 ± 10.51	270–360	300.0 ± 13.2	7.0 ± 5.0	0.40

The number of individuals largely varied between species (Table 3). A shark (MMU) and two skate species (LFU and LCI) were represented by only one and two specimens, respectively, while two species (SCA and GME) showed a very large number of individuals sampled (Table 3).

The size range of the species sampled showed different length intervals, haul mean depths, and CPUEs (Table 3). The CPUE calculated for GME was the highest among the studied species, and higher values were also observed for ESP and SCA compared to the other species (Table 3).

### 3.2. Intraspecific Variation of the Vitality Rate with Size

**Figure 3:**
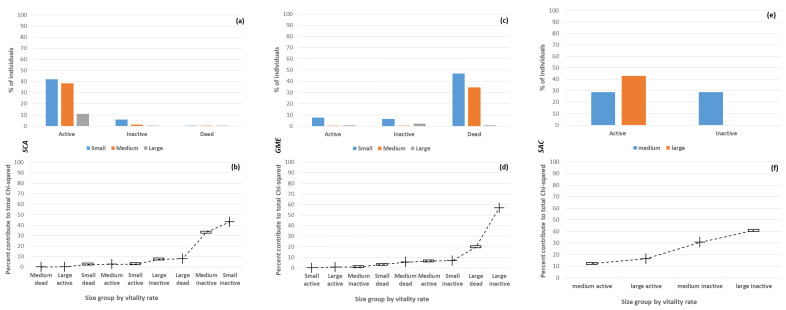
The observed percentage of the number of individuals by the vitality rate across size intervals (upper graph) and the increasing order of the percent contribution of groups to total within-species variation with respect to expected values (lower graph) observed in *Scyliorhinus canicula* (*SCA*, (**a**,**b**)), *Galeus melastomus* (*GME*, (**c**,**d**)), and *Squalus acanthias* (*SAC*, (**e**,**f**)) after trawl catch during a fishery-independent trawl survey in the Asinara Gulf during June–October 2022. Plus and minus labels on the data indicate observed values higher and lower than expected, respectively.

**Figure 4:**
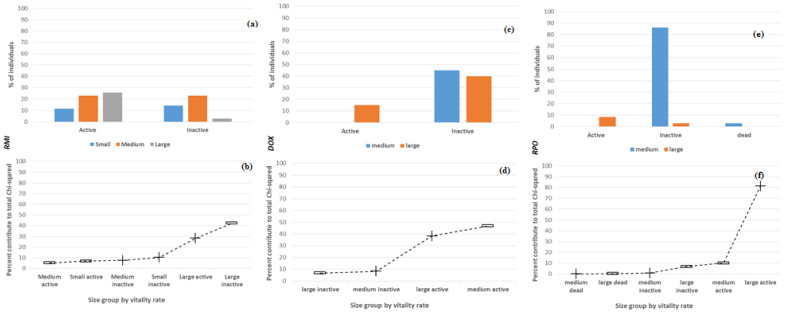
The observed percentage of the number of individuals by the vitality rate across size intervals (upper graph) and increasing order of the percent contribution of groups to total within-species variation with respect to expected values (lower graph) observed in *Raja miraletus* (*RMI*, (**a**,**b**)), *Dipturus oxyrinchus* (*DOX*, (**c**,**d**)), and *R. polystigma* (*RPOL*, (**e**,**f**)) after trawl catch during a fishery-independent trawl survey in the Asinara Gulf during June–October 2022. Plus and minus labels on the data indicate observed values higher and lower than expected, respectively.

*SCA*, *GME*, *SAC*, *RMI*, *DOX*, and *RPO* showed significant intraspecific differences (Table 4) between the observed (Figure 3a,c,e and Figure 4a,c,e respectively) and expected values of the abundance of individuals by vitality rate across size groups. In particular, inactive status was well represented in small specimens of *SCA* (Figure 3b) and in large individuals of *GME* (Figure 3d) relative to the other size vitality rate groups within each species.

The large specimens of *SAC* were in a better vitality condition than those of medium size (Figure 3f). A similar pattern was observed in *RMI* (Figure 4b), *DOX* (Figure 4d), and *RPO* (Figure 4f). In these species, the number of large inactive specimens was significantly lower than that of large active skates, and vice versa, in as far as the number of inactive small and/or medium specimens compared to active ones.

*RPO* was the only skate species where we observed dead individuals (Figure 4e). The general size-related pattern described above was also observed in the other species (*ESP*, *RMO*, and *RBR*). However, the differences in the relative abundance of individuals by vitality rate between the size groups (Table 4, Figure S2a–e, respectively) were not significant.

Within the species excluded from the analysis, we observed only active individuals in *LCI* and *MMU* and one active and one inactive specimen in *LFU*.

### 3.3. Interspecific Variation of the Vitality Rate

**Figure 5:**
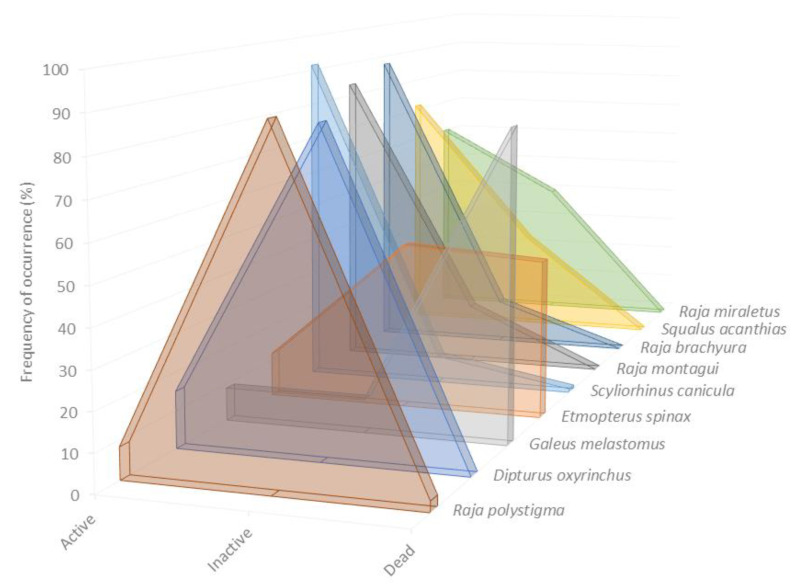
Vitality rates (active, inactive, and dead) after trawl catch in nine species of elasmobranchs sampled during a fishery-independent trawl survey in the Asinara Gulf during June–October 2022.

The relative abundance of individuals by the vitality rate was significantly different across the species (Friedman ANOVA: Χ^2^ = 6.23, N = 9, d. f. = 2; *p* < 0.05) and differently ranked within each species (mean rank = 0.26; Kendall Tau = 0.35). We distinguished three groups of species based on those with higher values for active (*SCA*, *RMI*, *SAC*, *RMO,* and *RBR*), inactive (*RPO* and *DOX*) and dead specimens (*ESP* and *GME*), respectively, compared to the occurrence of the other vitality rates (Figure 5).

In the post-hoc comparison of the groups (9 × 3 contingency table, Χ^2^ = 1530.09, d. f. = 16; *p* < 0.001), most of the contribution to the total variation was due to dead individuals of *GME*, which were much more numerous than expected (Figure S3). Dead and active *SCA* were less and more abundant than expected, with them being second in the contribution to the total variation (Figure S3). The third, fourth, and fifth contributions came from active individuals of *GME* (less than expected) and inactive individuals of *RPO* and *DOX* (more than expected) (Figure S3), respectively. After the sixth contribution (inactive *ESP*, more than expected), the contribution of species by the vitality rate to the total variation levelled at negligible decreasing values (Figure S3).

### 3.4. Factors Influencing the Vitality Rate

**Figure 6:**
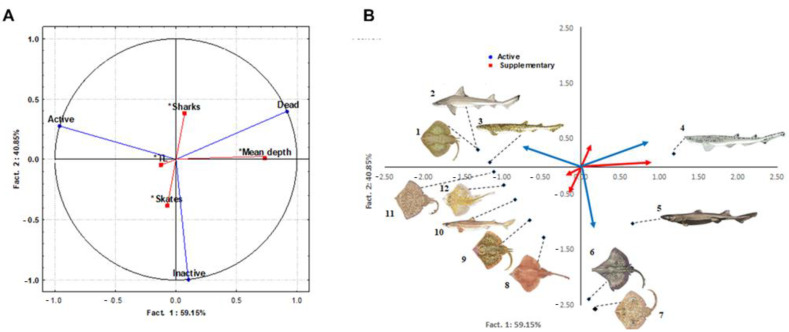
(**A**): Projection of active (active, inactive, and dead, as blue vectors) and supplementary (red asterisked vectors (*): catch mean depth, fish size as TL (total length), and skates and sharks, as taxonomical groups) variables on 1 × 2 factorial plane of principal component analysis used to investigate the differences in the vitality rates observed in twelve species of demersal elasmobranchs after trawl catch. (**B**): Projection of weighed mean of species coordinates on a 1 × 2 factorial plane together with vector variables. Note that factorial planes of the variables and cases refer to different coordinate systems such that the representation of vector variables in the cases’ plane provides qualitative information. Note that the length of supplementary vectors and the angle between them and active vectors are directly and inversely proportional to the average correlation between supplementary vectors and extracted components and between active and supplementary vectors, respectively. The species icons refer to *Leucoraja circularis* (1), *Mustelus mustelus* (2), *Scyliorhinus canicula* (3), *Galeus melastomus* (4), *Etmopterus spinax* (5), *Dipturus oxyrinchus* (6), *Raja polystigma* (7), *Leucoraja fullonica* (8), *R. miraletus* (9), *Squalus acanthias* (10), *R. brachyura* (11), and *R. montagui* (12) sampled during a fishery-independent trawl survey in the Asinara Gulf during June–October 2022. The species icons are modified from Barone M., Mazzoldi C., and Serena F. (2022) [79].

PCA extracted two principal components accounting for about 99.9% of the total variance. The four supplementary variables showed different correlations with the abundance of individuals by the vitality rate (active variables) in the species sampled (Figure 6A; Table 5). Catch mean depth was the most important factor, with it having the highest correlation with dead (direct) and active (inverse) individuals compared to the other supplementary variables (Table 5). The correlation between the morpho-taxonomical groups and the vitality rates of ‘dead’ and ‘inactive’ indicated sharks are directly related to ‘dead’ and inversely to ‘inactive’ rates and skates vice versa. Finally, fish size was inversely related to ‘dead’ and directly to ‘active’ vitality rates. However, the correlation between fish size and the vitality rate was the lowest observed between the active and supplementary variables.

Regarding the pairwise correlation between supplementary variables, fish size was inversely and directly correlated with sharks and skates, respectively, whereas the catch mean depth showed the opposite pattern (Table 5).

The species associated with an ‘active’ vitality rate, a low catch mean depth, and a large size were *SCA*, *MMU*, *SAC*, *RMO*, *RBR*, *RMI*, and *LCI* (Figure 6B). The species associated with an ‘inactive’ rate and an intermediate haul mean depth and size were *RPO* and *DOX*, with *LFU* representing a borderline species between the present and the former species group (Figure 6B). The last group of species (*ESP* and *GME*) was associated with a ‘dead’ vitality rate, a high mean depth, and a small size (Figure 6B).

Of the two main components extracted, the most important primarily represented the effect of variation in capture depth and, secondarily, the effect of fish size on the vitality rate of the sampled species (horizontal factor 1, Figure 6A,B, Table S1). The second component primarily explained the effect of species type and very weakly accounted for the effect of fish size (vertical factor 2, Figure 6A,B, Table S1).

## 4. Discussion

The present study demonstrates that the vitality rate of the species sampled varied as a function of inhabited depth, species type, and fish size, after demersal trawl catch in a fishery-independent experiment. On the one hand, the standardized and short tow duration adopted does not allow for comparison with other laboratory studies to mimic trawl capture. They demonstrated that fish stress associated with such capture is, of course, related to tow duration, but also to crowding and exposure to air [80,81,82]. However, the patterns of variation in the vitality rates that resulted in the present work have been described similarly in several discard species, including elasmobranchs, in response to capture by trawl or different gear [83,84,85].

Since the assemblage of the species sampled is well representative of the shark and rays’ diversity along with their bathymetrical distribution in the Mediterranean, the present standardized experiment allows for the comparison of the AVM between such different species.

The deepwater species sampled appeared as the species most affected by stress due to capture of trawling activities. The black-mouth catshark, the velvet belly, and the long-nosed skate were caught with CPUEs that are in line with the fishing yield known for these species within their expected bathymetrical range [86,87,88]. The high number of dead and inactive specimens observed in deepwater species may result from the interaction between factors related to species’ biological traits and variation in parameters connected to the capture of the sampled species [49].

Within the bathymetrical range explored in the area, seawater pressure, temperature, and light intensity are strongly different related to the vessel deck at the sea surface, spanning between 1 and 60 atm, between 14 °C and 40 °C (during the sampling period), and between 15 × 10^4^ and 150 lux, respectively [89]. The oceanographic parameters of the Mediterranean deepwater masses are rather constant. The basin is homoeothermic below 200 m depth due to the pump–heat effect between the basin and the Atlantic Ocean waters [90]. Additionally, the variation in seawater pressure and illumination with increasing depth is easily predictable at a given depth with a negligible error interval [91].

The abrupt change in the environmental light intensity could cause irreversible retinal damage in deepwater species, such as partial or total blindness of the caught individuals due to the potential burning of the retinal cellular epithelium [92]. Further studies are needed to study the poorly investigated effect of abrupt variation in light intensity on the vitality rate of elasmobranchs, both on the vessel and, mostly, upon release into the sea when the retinal damage might compromise survivorship in the natural environment.

Differently, large variations in water pressure [77,83,84,85] and temperature [93,94,95] are already known to induce dramatic physiological stress in elasmobranchs and bony fish. These two factors can even interact to favor embolic phenomenon that can resolve with the death of individuals during capture [96]. This serious problem is due to the animal’s inability to compensate for internal body pressure versus external water pressure when a change occurs too quickly for the former to adapt efficiently [97].

In addition, in the present experiment, the high number of dead and inactive specimens observed in deepwater species can result from an embolic phenomenon due to the too rapid and combined effect of increasing temperature and decreasing water pressure with decreasing depth during hauling operations [96]. This applies to the net hauling time observed (45 min on average) compared to variations in seawater pressure (1 to 30, max 60 atm) and temperature (14 °C to 35–40 °C on the hottest sampling days) between the sea floor and the vessel deck during the sampling operations at deep water stations. A clue to the embolic phenomenon was the presence of edema along the body of deepwater skate *D. oxyrinchus* and black-mouth catshark *G. melastomus*. These injuries were more evident in these species as they are lighter in color pattern compared to the other deepwater species caught. Therefore, we cannot exclude embolic signs in the darker velvet belly.

The skin thickness and composition of the catch mass also have to be considered among factors influencing the vitality rate [75,98,99,100,101], particularly in the velvet belly and the long-nosed skate. The thin skins of these species [101] might expose them to potential injuries due to the compresence in the catch mass of harmful species, as presently observed for decapod crustaceans, such as the Norway lobster *N. norvegicus*, the rose shrimp *P. longirostris,* and the red and violet shrimps *A. antennatus* and *A. foliacea*. Although no particular external damage was found on the bodies of the sampled specimens, we cannot rule out the possibility of injuries to the internal organs. Thin skin associated with potentially harmful species in the catch mass can reduce the vitality rate in elasmobranch species [98,99,100].

The case of the blackmouth catshark suggested that thermic shock and embolic phenomenon is the more likely cause of death in this species, as it showed the largest number of dead specimens and signs of edema, despite its more robust skin compared to the other deepwater species.

On the other hand, coastal species exhibited a higher and lower number of active and inactive individuals, respectively (i.e., a better vitality condition with respect to deepwater species). In fact, the probability of embolic phenomenon and the temperature variation is strongly reduced with decreasing fishing depth [77,83,84,85,93,94,95]. Furthermore, the effect of potentially harmful species in the catch mass (sea urchins, octopuses, and crabs) is buffered by the more robust skin of coastal species compared to deep water species [100,101], as a robust skin lowers AVM in elasmobranchs [98,99,100]. In addition, for coastal species, the mean depth of the catch and CPUEs were in line with the existing information for Mediterranean waters [73].

The relationship between species type and vitality rate indicated that skates are more resistant to catch than sharks. This result can be explained by observing the difference in the relative abundance of inactive individuals between the two species groups. Skates appeared to have more individuals in an inactive state after the catch than sharks, particularly the deep- and shelf-water skates *D. oxyrinchus* and *R. polystigma*, respectively. Given the fewer dead specimens observed in skates, this could be linked to a higher recovery buffer skates may have and take advantage of compared to sharks. The better respiratory efficiency of the spiracles in skates compared to sharks may represent one of the variables to consider [102]. Developed spiracles of skates are adaptive to benthic habit in elasmobranch species, facilitating respiration, in particular when the animal stands in a non-swimming mode on the seafloor [102]. Spiracles could similarly function during the catch, which dramatically limits the body movements of the animal. Indeed, species with assisted gill ventilation, such as buccal pump ventilation, appear more resistant to catch than species needing ram ventilation to breathe efficiently [103].

Size played an important role in influencing the vitality rate, as larger individuals were generally more resistant to capture than medium ones and, in particular, they were more resistant than smaller specimens. It is well known that species resistance to a disturbance, fishing catch included, increases with size, especially in k-selected species such as elasmobranchs [76,83,84,93,103,104,105]. Confirming this, such a general trend appeared more pronounced in skates, due to their larger size, than in sharks, as evidenced by the intraspecific variation of vitality rate with size.

As corollary data, the present scientific survey found a slightly different faunal list for the elasmobranchs present in the waters of the Northern Sardinia, as compared with information available for the north-western Mediterranean [106]. It is worth noting the presence of two species of the genus *Leucoraja* sp., such as *L. fullonica* and *L. circularis*, which are considered as rare species in the Mediterranean Sea [72,107]. Despite the careful species identification carried out at the meristic level, caution is required in validating species identity, as coupled genetic assessments were not implemented on the sampled species. Species misidentification is frequent in elasmobranchs, such as in the genus *Squalus* sp. [108,109] and in the Rajidae family due to the general high variability of their morphological characteristics, changes in color patterns, shapes, and relative body proportions during ontogeny [110], and hybridization with sibling species [111]. For instance, the presence of *S. acanthias* and *R. montagui* is novel in the northern Mediterranean Sea [106]. On the other hand, the distributions of an increasing number of marine species, elasmobranchs included, are rapidly changing worldwide in response to the variation of oceanographic parameters inducing those caused by climate change [112]. Therefore, faunal lists are far to be static nowadays, especially at the local level where new species can be present yet are not expected. However, future meristic analyses coupled with genetic studies will be necessary to confirm the identity of the species found in the investigated area.

The information provided by this work can help refine best practices to reduce direct and indirect fishing mortality of the studied species in trawling activities. For instance, the data suggest that sharks, and small specimens in general, are priority items in after-capture treatment for physiological recovery and release at sea operations, as they are less resistant than skates to trawl catch. In addition, deepwater species could benefit from a reduced trawl hauling speed to limit thermic and barometric shocks, which are probably responsible for the significant number of dead specimens observed in these species.

Future research will focus on refining the estimate of vitality rates of the sampled specimens before releasing them at sea. For instance, it will be necessary to assess the individual vitality rate both after capture, as carried out in the present study, and before the specimens are released at sea after a period of physiological recovery in oxygenated tanks. The duration of the recovery period will also need to be standardized by species and/or size, as size and species affect the vitality rate after capture, as demonstrated by the present work. This will optimize the great logistic effort needed for the most abundant/large-sized species, i.e., to organize many different sized tanks to allow individual observations and the coupled assessments of vitality rates of each caught and released specimen.

Release at sea will also be improved to counteract the depredation activity observed on released sharks and skates carried out by seagulls. Simultaneous and safe underwater release of samples at sea might abate depredation by sea birds, contrasting their ability to catch their prey up to several meters underwater.

Additionally, different tagging methods are available to monitor post release mortality in elasmobranchs [49,85], and several bycatch mitigation options can be implemented in trawl fishery, such as exclusion grids or other techniques and devices [113,114,115,116].

## 5. Conclusions

Overall, the present work has provided evidence that the vitality rate of the studied species results from the intermingled effect of the inhabited depth, species type, and fish size, with deepwater small-sized sharks being the most affected by stress due to trawling capture. On the contrary, large and coastal species, particularly skates, appeared to be more resistant to trawl activities.

Due to their high resistance to capture, elasmobranchs can benefit from being released back into the sea after capture, especially if they are healthy. Their survival after release can complement bycatch avoidance techniques in trawl fisheries. A multilevel approach to mitigate the problem of elasmobranch bycatch in trawling activities can increase the resilience capacity of the elasmobranch populations suffering from the impact of this fishery activity.

## Figures and Tables

**Table 1:** The scale of the vitality rates used to assess the resistance to capture of twelve elasmobranch species through a fishery-independent trawl survey in the Asinara Gulf during June–October 2022.

Level of Vitality after Trawl Capture	Description
Dead	No body movement nor any contraction of spiracles, gills, and the mouth
Inactive	Weak body movements with some inconstant contractions of spiracles, gills, and the mouth
Active	Strong and active body movements with constant contractions of spiracles, gills, and the mouth

**Table 2:** Species code (letters denote the initial letter and the first two letters of the genus and species name, respectively) arranged in alphabetical order and the length range of the size groups (small, medium, and large) used in the analysis of the vitality rates variation with size in twelve species of elasmobranchs after capture through a fishery-independent trawl survey in the Asinara Gulf during June–October 2022. The underlined species were excluded from quantitative analyses. Species codes are based on the initial letter and the first two letters of the genus and the species name, respectively.

Species	Species Code	Length Range of Size Groups (cm)		
		Small	Medium	Large
*Dipturus oxyrinchus*	*DOX*	na	24–43	44–62
*Etmopterus spinax*	*ESP*	12–22	23–32	33–42
*Galeus melastomus*	*GME*	10–23	24–36	37–49
*Leucoraja circularis*	*LCI*	na	Na	na
*Leucoraja fullonica*	*LFU*	na	Na	na
*Mustelus mustelus*	*MMU*	na	Na	na
*Raja brachyura*	*RBR*	na	24–40	41–57
*Raja miraletus*	*RMI*	13–22	23–32	33–41
*Raja montagui*	*RMO*	na	9–28	29–47
*Raja polystigma*	*RPO*	na	15–35	36–55
*Scyliorhinus canicula*	*SCA*	10–23	24–37	38–50
*Squalus acanthias*	*SAC*	na	45–58	59–72

**Table 4:** Chi-square (Χ^2^) values and related statistics (d.f.: degrees of freedom of the corresponding contingency tables, p: statistical significance, * *p* < 0.1, ** *p* < 0.05, *** *p* < 0.001) of the within-species comparison of the individuals’ abundance by size group (small, intermediate, and large or intermediate and large) and by vitality rate (active, inactive, and dead or active and inactive) for each of the nine elasmobranch species collected through a fishery-independent trawl survey in the Asinara Gulf during June–October 2022. Species codes *DOX*, *ESP*, *GME*, *LCI*, *LFU*, *MMU*, *RBR*, *RMI*, *RMO*, *RPO*, *SAC*, and *SCA* refer to *Dipturus oxyrinchus*, *Etmopterus spinax*, *Galeus melastomus*, *Leucoraja circularis*, *L. fullonica*, *Mustelus mustelus*, *Raja brachyura*, *R. miraletus*, *R. montagui*, *R. polystigma*, *Scyliorhinus canicular*, and *Squalus acanthias*, respectively.

Species	Χ^2^	d. f.	*p*
*DOX*	2.89	1	**
*ESP*	4.43	4	ns
*GME*	150.29	4	***
*RBR*	0.00	1	ns
*RMI*	5.32	2	**
*RMO*	1.71	1	ns
*RPO*	26.19	2	***
*SCA*	22.46	4	***
*SAC*	2.10	1	*

**Table 5:** Correlation matrix of active and supplementary (asterisked) variables used in the principal component analysis aimed at detecting factors determining differences in the at-vessel vitality rate between nine species of demersal elasmobranchs sampled during a fishery-independent trawl survey in the Asinara Gulf during June–October 2022. Active and supplementary variables are rates of vitality after trawl catch (active, inactive, and dead) and catch mean depth, fish size (TL: total length), and two taxonomical groups (skates and sharks), respectively.

Variables	Dead	Inactive	Active	* TL	* Mean Depth	* Sharks	* Skates
Dead	1.00	−0.30	−0.77	−0.14	0.68	0.22	−0.22
Inactive	−0.30	1.00	−0.37	0.03	0.07	−0.36	0.36
Active	−0.77	−0.37	1.00	0.11	−0.70	0.03	−0.03
* TL	−0.14	0.03	0.11	1.00	−0.09	−0.18	0.18
* Mean depth	0.68	0.07	−0.70	−0.09	1.00	0.21	−0.21
* Sharks	0.22	−0.36	0.03	−0.18	0.21	1.00	−1.00
* Skates	−0.22	0.36	−0.03	0.18	−0.21	−1.00	1.00

## Data Availability

Data are available from the corresponding author upon reasonable request.

## Supplementary Materials

The following supporting information can be downloaded at: https://www.mdpi.com/article/10.3390/biology12030363/s1. Figure S1: Images of the shark species sampled during a fishery independent trawl survey in the Asinara Gulf during June-October 2022. (a) *Etmopterus spinax*, (b) *Squalus acanthias*, (c) *Galeus melastomus*, (d) close-up of a specimen of *G. melastomus* with an oedema over the skin of the lower jaw, (e) *Mustelus mustelus*, and (f) *Scyliorhinus canicula* (ROV footage); Figure S2: Observed percentage of the number of individuals by vitality’s rate across size intervals (upper graph) and increasing order of the percent contribution of groups to total within-species variation with respect to expected values (lower graph) observed in *Etmopterus spinax* (ESP, a, b), *Raja montagui* (RMO, c, d) and *Raja brachyura* (RBR, e) after trawl catch during a fishery independent trawl survey in the Asinara Gulf during June–October 2022. Plus and minus labels on data indicate observed values higher or lower than expected, respectively; Figure S3: Increasing order of the percent contribution of groups (species by vitality rate) to the total interspecific variation in the comparison between the observed and expected values of the individuals’ abundance by group in nine species of elasmobranchs sampled during a fishery independent trawl survey in the Asinara Gulf during June–October 2022. Plus and minus labels on data indicate observed values higher or lower than expected, respectively. Table S1: Factor loadings of active and supplementary (asterisked) variables as resulted from the Principal Component Analysis applied to detect differences in the vitality rate of twelve species of elasmobranchs sampled during a fishery independent trawl survey in the Asinara Gulf during June–October 2022.
