# Supplementary material for: Gradients of Variation in the At-Vessel Mortality Rate between Twelve Species of Sharks and Skates Sampled through a Fishery-Independent Trawl Survey in the Asinara Gulf (NW Mediterranean Sea)
Source: Biology (Basel). 2023 Feb 24;12(3):363. doi: 10.3390/biology12030363 (PMC10044918; doi:10.3390/biology12030363)
Supplement: Supplementary file 1 [file biology-12-00363-s001.zip › Figure S2.pdf]

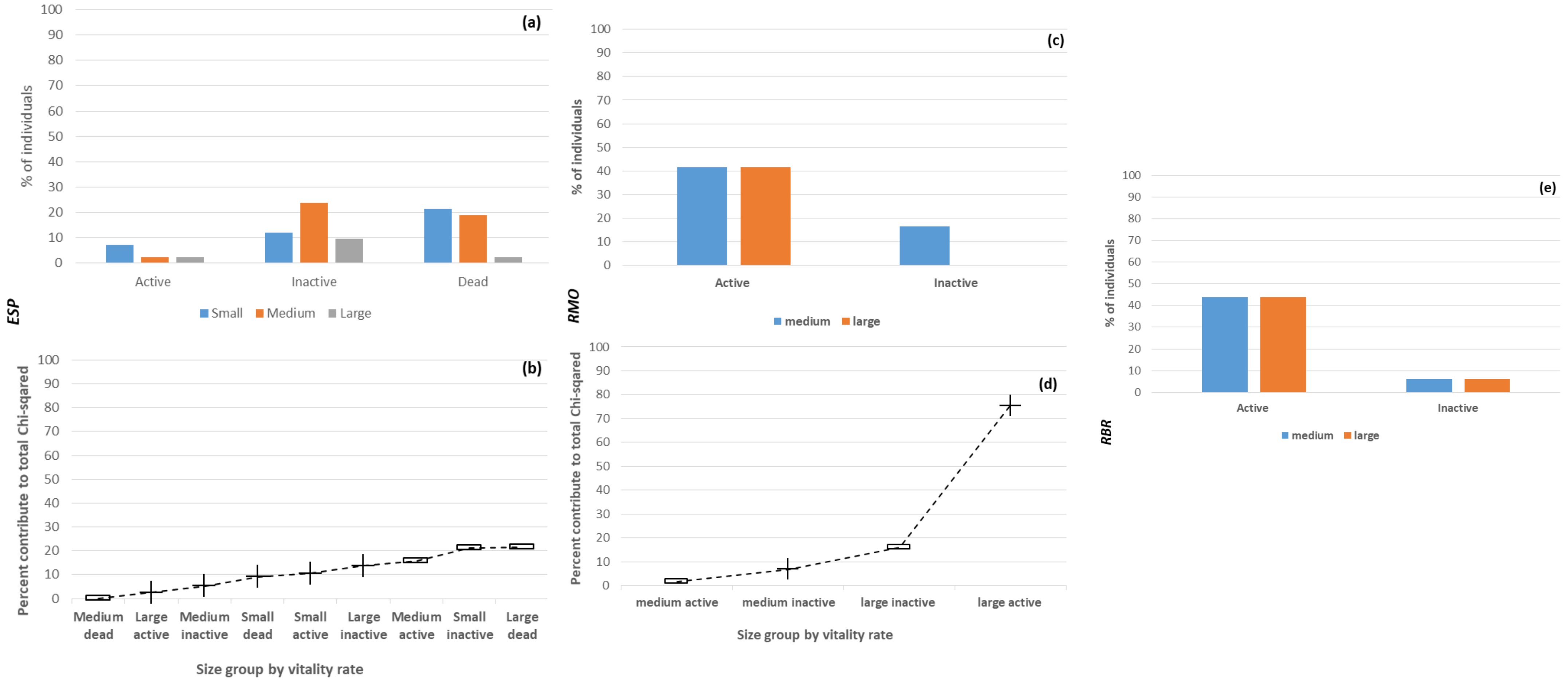

Figure S2. Observed percentage of the number of individuals by vitality's rate across size intervals (upper graph) and increasing order of the percent contribution of groups to total within-species variation with respect to expected values (lower graph) observed in *Etmopterus spinax* (*ESP*, a, b), *Raja montagui* (*RMO*, c, d) and *Raja brachyura* (*RBR*, e) after trawl catch during a fishery independent trawl survey in the Asinara Gulf during June-October 2022. Plus and minus labels on data indicate observed values higher or lower than expected, respectively.
