# Supplementary material for: Gradients of Variation in the At-Vessel Mortality Rate between Twelve Species of Sharks and Skates Sampled through a Fishery-Independent Trawl Survey in the Asinara Gulf (NW Mediterranean Sea)
Source: Biology (Basel). 2023 Feb 24;12(3):363. doi: 10.3390/biology12030363 (PMC10044918; doi:10.3390/biology12030363)
Supplement: Supplementary file 1 [file biology-12-00363-s001.zip › Figure S3.pdf]

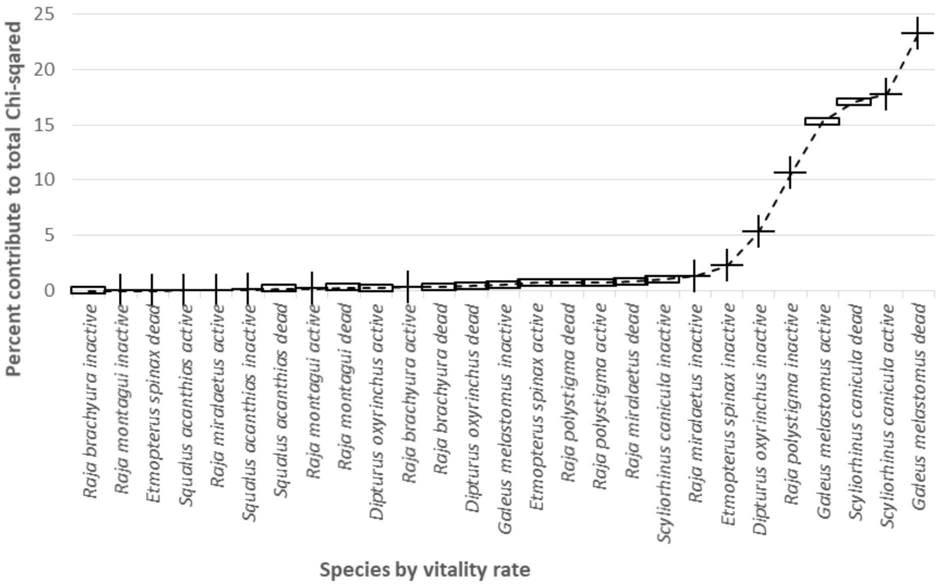

Figure S3. Increasing order of the percent contribution of groups (species by vitality rate) to the total interspecific variation in the comparison between the observed and expected values of the individuals' abundance by group in nine species of elasmobranchs sampled during a fishery independent trawl survey in the Asinara Gulf during June-October 2022. Plus and minus labels on data indicate observed values higher or lower than expected, respectively.
