# Supplementary material for: Gradients of Variation in the At-Vessel Mortality Rate between Twelve Species of Sharks and Skates Sampled through a Fishery-Independent Trawl Survey in the Asinara Gulf (NW Mediterranean Sea)
Source: Biology (Basel). 2023 Feb 24;12(3):363. doi: 10.3390/biology12030363 (PMC10044918; doi:10.3390/biology12030363)
Supplement: Supplementary file 1 [file biology-12-00363-s001.zip › Table S1.pdf]

Table S1. Factor loadings of active and supplementary (asterisked) variables as resulted from the Principal Component Analysis applied to detect differences in the vitality rate of twelve species of elasmobranchs sampled during a fishery independent trawl survey in the Asinara Gulf during June-October 2022.

	**Extracted Factors**		
**Variable**	**1**	**2**	**3**
Dead	0.916034	0.401043	-0.006758
Inactive	0.103955	-0.994571	-0.004641
Active	-0.961552	0.274534	-0.006940
*TL	-0.129174	-0.044903	-0.016858
*Mean depth	0.736540	0.010067	-0.003280
*Sharks	0.079007	0.375993	0.002949
*Skates	-0.079007	-0.375993	-0.002949
