# Supplementary material for: Gradients of Variation in the At-Vessel Mortality Rate between Twelve Species of Sharks and Skates Sampled through a Fishery-Independent Trawl Survey in the Asinara Gulf (NW Mediterranean Sea)
Source: Biology (Basel). 2023 Feb 24;12(3):363. doi: 10.3390/biology12030363 (PMC10044918; doi:10.3390/biology12030363)
Supplement: Supplementary file 1 [file biology-12-00363-s001.zip › Figure S1.pdf]

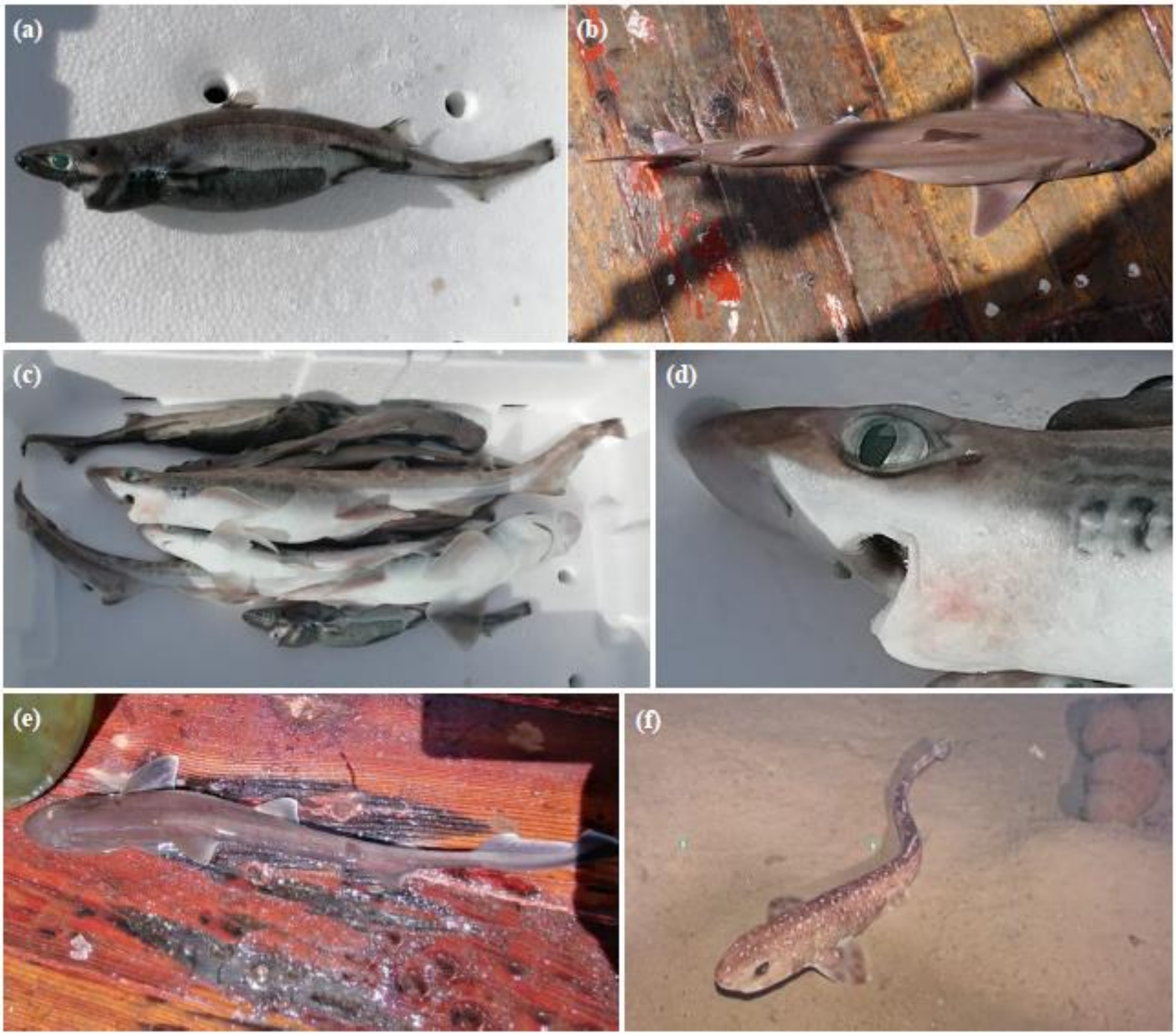

Figure S1. Images of the shark species sampled during a fishery independent trawl survey in the Asinara Gulf during June-October 2022. (a) *Etmopterus spinax*, (b) *Squalus acanthias*, (c) *Galeus melastomus*, (d) close-up of a specimen of *G.melastomus* with an oedema over the skin of the lower jaw, (e) *Mustelus mustelus*, and (f) *Scyliorhinus canicula* (ROV footage).
